# Anticancer Drugs for Intra-Arterial Treatment of Colorectal Cancer Liver Metastases: In-Vitro Screening after Short Exposure Time

**DOI:** 10.3390/ph14070639

**Published:** 2021-07-01

**Authors:** Audrey Fohlen, Karim Bordji, Eric Assenat, Céline Gongora, Céline Bazille, Jérémy Boulonnais, Mikaël Naveau, Cécile Breuil, Elodie A. Pérès, Myriam Bernaudin, Boris Guiu

**Affiliations:** 1 UNICAEN, CEA, CNRS, ISTCT/CERVOxy Group, GIP CYCERON, Normandie University, 14000 Caen, France; bordji@cyceron.fr (K.B.); bazille-c@chu-caen.fr (C.B.); boulonnais@cyceron.fr (J.B.); peres@cyceron.fr (E.A.P.); bernaudin@cyceron.fr (M.B.); 2 Urodigestive Imagery and Interventional Radiology Department, University Hospital of Caen, CEDEX, 14000 Caen, France; 3 Medical Oncology Department, Montpellier School of Medicine, Saint-Eloi University Hospital, 80 Avenue Augustin Fliche, 34295 Montpellier, France; e-assenat@chu-montpellier.fr; 4 IRCM, Montpellier Cancerology Research Center, INSERM U1194, Montpellier University, Montpellier Regional Institute of Cancer, 34298 Montpellier, France; celine.gongora@inserm.fr; 5 Department of Pathology, University Hospital of Caen, CEDEX, 14000 Caen, France; 6 UNICAEN, CNRS, UMS 3408, GIP CYCERON, Normandie University, 14000 Caen, France; naveau@cyceron.fr; 7 Pharmacy Department, University Hospital of Caen, CEDEX, 14000 Caen, France; breuil-c@chu-caen.fr; 8 Radiology Department, Montpellier School of Medicine, Saint-Eloi University Hospital, 80 Avenue Augustin Fliche, 34295 Montpellier, France; b-guiu@chu-montpellier.fr

**Keywords:** antineoplastic agents, colorectal neoplasms, hepatic artery, liver neoplasm, in vitro screening

## Abstract

To treat colorectal liver metastases, intra-arterial chemotherapies may complete therapeutic arsenal. Drugs using intra-arterially are very heterogeneous. The aim of this study was to select the most efficient drug on a panel of colorectal cancer (CRC) cell lines (Caco-2, HCT 116, HT 29, SW 48, SW 480, SW 620) exposed for 30 min to 12 cytotoxic agents (doxorubicin, epirubicin, idarubicin, 5-FU, raltitrexed, gemcitabine, cisplatin, oxaliplatin, mitomycin C, irinotecan, streptozocin, paclitaxel) at different concentrations. The effect on cell viability was measured using the WST-1 cell viability assay. For each drug and cell line, the IC_50_ and IC_90_ were calculated, which respectively correspond to the drug concentration (mg/mL) required to obtain 50% and 90% of cell death. We also quantified the cytotoxic index (CyI_90_ = C Max/IC_90_) to compare drug efficacy. The main findings of this study are that idarubicin emerged as the most cytotoxic agent to most of the tested CRC cell lines (Caco-2, HT29, HCT116, SW620 and SW480). Gemcitabine seemed to be the most efficient chemotherapy for SW48. Interestingly, the most commonly used cytotoxic agents in the systemic and intra-arterial treatment of colorectal liver metastasis (CRLM) (oxaliplatin, 5-FU, irinotecan) showed very limited cytotoxicity to all the cell lines.

## 1. Introduction

Colorectal cancer (CRC) represents the third most common type of cancer in the world and the second in terms of cancer mortality [1]. Synchronous colorectal liver metastases (CRLM) occur in up to 50% of patients [2]. Complete surgical resection represents the best treatment for long-term survival. Even though less than 25% of patients are initially eligible for liver surgery [3], liver metastases may become resectable after chemotherapy.

Nowadays, systemic treatments in metastastic CRC combine 2 or 3 drugs such as FOLFOX (fluorouracil, folinic acid, oxaliplatin), FOLFIRI (fluorouracil, folinic acid, irinotecan), or FOLFIRINOX (fluorouracil, folinic acid, oxaliplatin, and irinotecan) with targeted therapies (bevacizumab, cetuximab, or panitumumab) depending on the RAS status [4,5,6,7]. Studies report a correlation between response rate and secondary resection rate [4,5,6,7]. Nevertheless, 30–40% of RAS-wt patients are non-responders to anti-EGFR treatments [8]. Moreover, long-term toxicity for systemic treatment may be observed [9]. For RAS mutated tumors (Kras or Nras), which represent about 50–60% of CRC patients, available treatment lines are very limited.

Intra-arterial (IA) therapies have been developed for a long time to treat liver metastases [10,11,12,13,14]. Different IA approaches were proposed: hepatic arterial infusion of chemotherapy (HAIC), chemoembolization with lipiodol (conventional trans-arterial chemoembolization or c-TACE), and drug-eluting beads trans-arterial chemoembolization (DEB-TACE) [14]. All these techniques take advantage of the 99% arterial blood supply of liver tumors, whereas the non-tumoral parenchyma is mainly supplied by the portal vein. Consequently, high drug concentrations can be achieved within the tumor while limiting systemic exposure and subsequent side effects. IA therapies could act as salvage treatments in unresectable patients with insufficient response rates following standard IV regimens, and especially in RAS-mutated patients for whom the number of treatment lines is very limited.

Among IA techniques, HAIC has been the most widely investigated in CRLM, based on two different regimens: FUDR-based [15] or oxaliplatin-based [16,17] IA infusion. Despite an attractive rationale and promising response rates [18,19], a benefit in overall survival (OS) has never been demonstrated so far [19,20,21,22]. Technical difficulties with implanted pumps (for FUDR) or with the implantation of IA catheters have certainly impaired the efficacy of these approaches and strongly limited their widespread use [23,24].

Other IA techniques are easier to implement in routine clinical practice, but efforts should be made to optimize the treatment regimen. Surprisingly, drugs used for IA therapies vary a lot [25].

However, to our knowledge, drug selection has never been based on any cytotoxicity screening in CRC cell lines. Yet, it might be useless to reach higher intra-tumoral concentration thanks to IA injection if the drug is ineffective against the tumor cells. Varela et al. studied doxorubicin pharmacokinetics (PK) after intra-arterial treatment of hepatocellular carcinoma (HCC) [26] and showed that the peak drug concentration in serum was reached within 5 min after injection for both techniques, followed by a decrease in systemic release only 20 to 40 min after injection [26]. This led Boulin et al. to screen anticancer drugs on HCC cell lines after a short exposure time (30 min) to optimize drug selection for IA treatment [27]. They tested 11 drugs in vitro on 3 different cell lines and showed that the most frequently-used drug for TACE in HCC (i.e., doxorubicin) was not the best candidate compared with idarubicin, which displayed the greatest cytotoxicity profile [27]. The same group conducted further clinical studies showing promising results with idarubicin [28,29,30,31].

The present study aimed to screen drugs (currently or not currently used on CRC) on a panel of CRC cell lines with a short exposure time (30 min), in order to select the most efficient one to design further clinical trials of IA treatments for CRLM. The assessment of drug mechanism was however outside the scope of this study.

## 2. Results

### 2.1. Viability Curves for 12 Tested Cytotoxic Agents on 6 CRC Cell Lines (HCT116, HT29, Caco-2, SW48, SW480 and SW620)

#### 2.1.1. Topoisomerase II Inhibitor (Doxorubicin, Epirubicin and Idarubicin)

**Figure 1:**
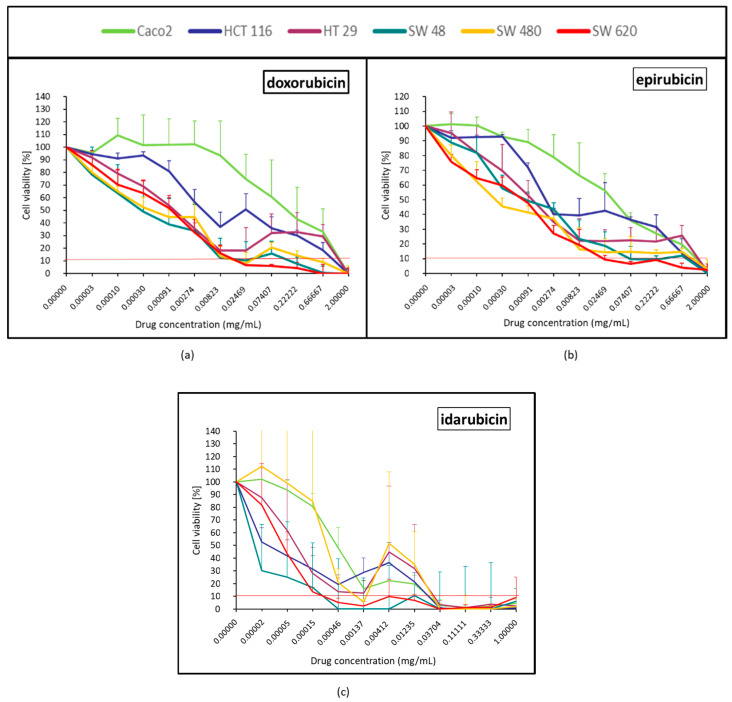
Colorectal cancer cell line viability curves (mean ± standard deviation) after a 30 min contact time with the 3 anthracyclines: doxorubicin (**a**), epirubicin (**b**), and idarubicin (**c**). The horizontal red line represents 10% of cell viability. The results are presented after normalization with the control cells (untreated condition).

Topoisomerase II inhibitors had a strong effect over SW620. Regarding doxorubicin and epirubicin, Caco-2, HCT116 and HT29 (at high concentrations for the latter) were the most resistant cell lines (Figure 1a,b). With doxorubicin and epirubicin, after 4 dilutions, a dose-effect was observed. Even at low concentrations, idarubicin had a strong effect, particularly over SW48 and SW620 (Figure 1c). No cell line exhibited any major resistance to idarubicin, except at very low concentrations for Caco-2 and SW480. For these 2 cell lines, we observed that 10% of cell viability was achieved after 7 dilutions of idarubicin (Figure 1c). With idarubicin, a moderate rebound effect occurred after 6 dilutions in most of the cell lines. For doxorubicin, epirubicin, and idarubicin, the viability curves of the cell line derived from the primary tumor (SW480) and metastasis of which (SW620) had quite a similar response profile.

#### 2.1.2. Anti-Metabolites Effect (5-FU, Raltitrexed and Gemcitabine)

**Figure 2:**
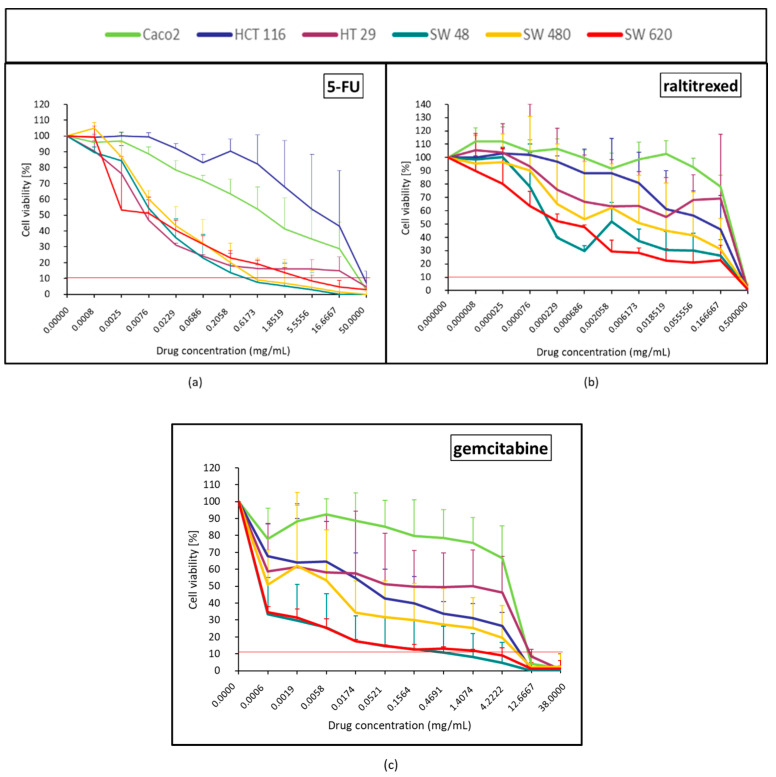
Colorectal cancer cell line viability curves (mean ± standard deviation) after a 30 min contact time with the 3 anti-metabolites: 5-FU (**a**), raltitrexed (**b**), and gemcitabine (**c**). The horizontal red line represents 10% of cell viability. The results are presented after normalization with the control cells (untreated condition).

Regarding 5-FU, HCT116 and Caco-2 were quickly resistant (Figure 2a). Compared with raltitrexed and gemcitabine, 5-FU was the most efficient over HT29, SW48, SW480, and SW620. With raltitrexed, cell viability is lowered to 10% only when it is used at maximum concentration (Figure 2b). Gemcitabine was mainly efficient over SW620 and SW48 (Figure 2c). A large plateau effect was observed with raltitrexed and gemcitabine for most of the tested concentrations (Figure 2b,c) and the cytotoxicity profile of these two drugs strongly differed from one cell line to another (Figure 2b,c). Caco-2 was the most resistant cell line with raltitrexed and gemcitabine (Figure 2b,c).

#### 2.1.3. Platinum Derivatives (Cisplatin and Oxaliplatin)

**Figure 3:**
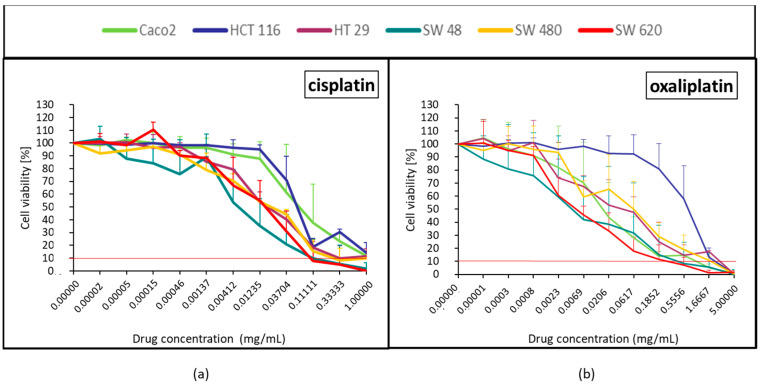
Colorectal cancer cell line viability curves (mean ± standard deviation) after a 30 min contact time with the 2 platinum derivatives: cisplatin (**a**) and oxaliplatin (**b**). The horizontal red line represents 10% of cell viability. The results are presented after normalization with the control cells (untreated condition).

A dose-effect relationship was strongly and quickly observed with cisplatin and oxaliplatin (Figure 3). With cisplatin, cell viability under 10% was only observed at Cmax and only in 2 cell lines (SW48 and SW620). Oxaliplatin quickly had no effect on HCT116 (Figure 3b).

#### 2.1.4. Alkylating Antibiotic (Mitomycin C)

**Figure 4:**
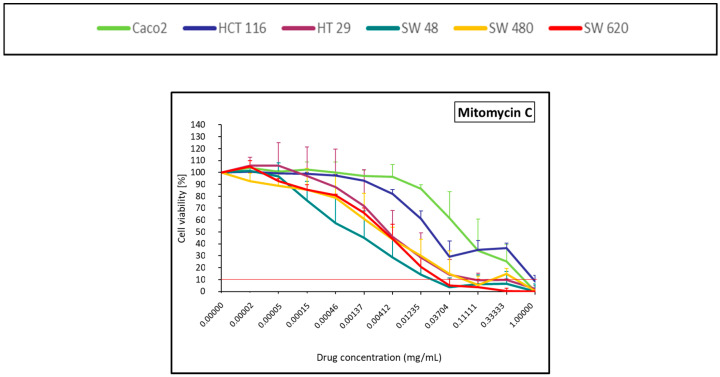
Colorectal cancer cell line viability curves (mean ± standard deviation) after a 30 min contact time with an alkylating antibiotic (mitomycin C). The horizontal red line represents 10% of cell viability. The results are presented after normalization with the control cells (untreated condition).

The alkylating antibiotic, mitomycin C, led to more than 90% of cell death from the first 3 dilutions for SW48, SW480, SW620, and HT29 (Figure 4). HCT 116 and Caco-2 displayed resistant profiles.

#### 2.1.5. DNA Topoisomerase I Inhibitor (Irinotecan)

**Figure 5:**
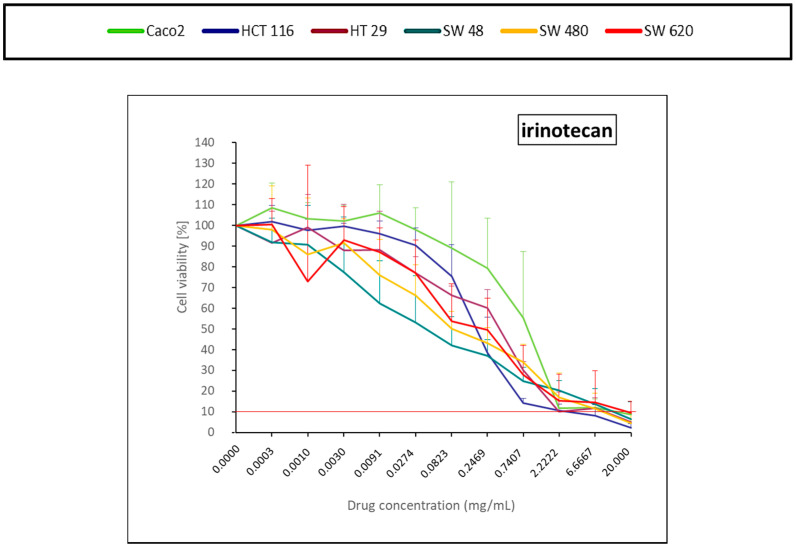
Colorectal cancer cell line viability curves (mean ± standard deviation) after a 30 min contact time with a DNA topoisomerase inhibitor (irinotecan). The horizontal red line represents 10% of cell viability. The results are presented after normalization with the control cells (untreated condition).

Irinotecan led to 90% of cell death at high concentrations (Figure 5), but was no longer cytotoxic from the fourth dilution, whatever the cell line (Figure 5).

#### 2.1.6. Antitumoral Antibiotic (Streptozocin)

**Figure 6:**
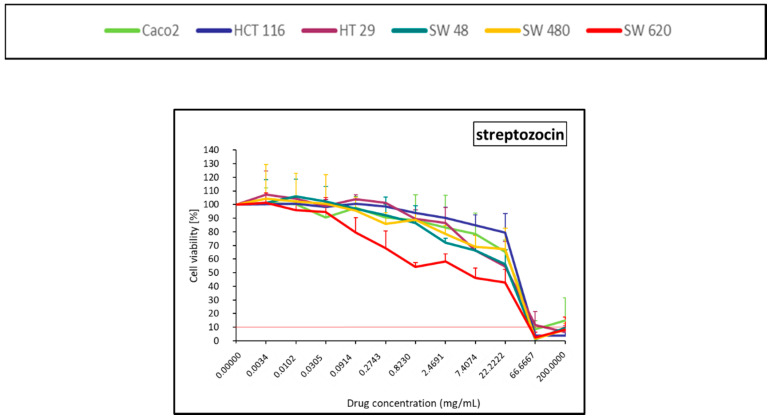
Colorectal cancer cell line viability curves (mean ± standard deviation) after a 30 min contact time with an antitumoral antibiotic (streptozocin). The horizontal red line represents 10% of cell viability. The results are presented after normalization with the control cells (untreated condition).

The antitumoral antibiotic, streptozocin, had almost no cytotoxicity effect, except at Cmax and at the first dilution (Figure 6).

#### 2.1.7. Taxane (Paclitaxel)

**Figure 7:**
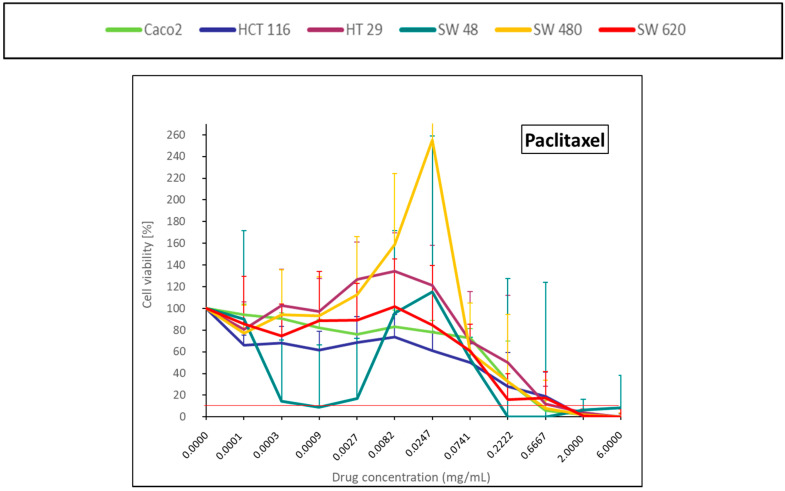
Colorectal cancer cell lines viability curves (mean ± standard deviation) after a 30-min contact time with a taxane (paclitaxel). The horizontal red line represents 10% of cell viability. The results are presented after normalization with the control cells (untreated condition).

The cell viability curve of taxane (paclitaxel) (Figure 7) showed a cell proliferation for intermediate concentration.

The statistical analysis (Anova and post-hoc test (LSD-Fisher)) compared cell viability for each drug; control versus any concentration is available in Supplementary Table S1.

### 2.2. IC_50_, IC_90_ and Cytotoxicity Index (CyI_90_) for Each CRC Cell Line According the 12 Antitumoral Agents

The cytotoxic agent concentrations inducing 50% and 90% of cell death (IC_50_ and IC_90,_ respectively) are represented in Table 1 and Table 2. Idarubicin is the cytotoxic agent with the lowest IC_50_ and IC_90_.

To compare the effect of the different agents over the studied cell lines, the cytotoxic index (CyI_90_) was determined for each agent and cell line by calculating the ratio of maximal concentration to IC_90_. The values are represented in Table 3. The highest value of CyI_90_ was obtained by idarubicin for all the studied cell lines, except for SW48, over which gemcitabine had the highest CyI_90_ (Table 3).

**Figure 8:**
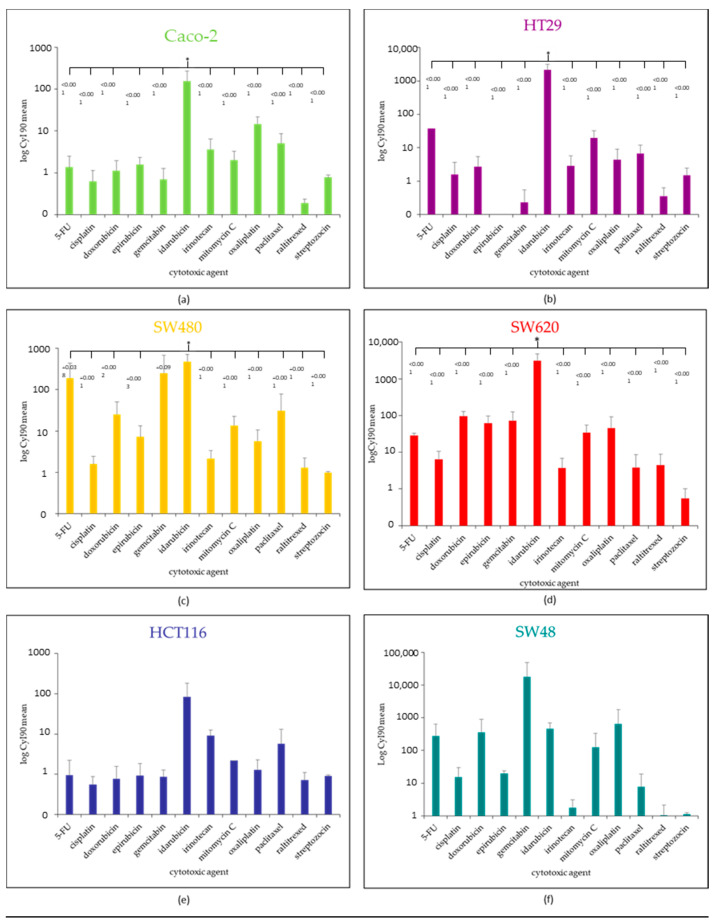
Bar plot representation of the cytotoxic indexes (mean ± standard deviation) for the 6 colorectal cancer cell lines: Caco-2 (**a**), HT29 (**b**), SW480 (**c**), SW620 (**d**), HCT116 (**e**), SW48 (**f**). The significant *p*-values of the post-hoc tests (LSD Fisher) were reported for cell lines with significant one-way ANOVA (*p* < 0.05).

For the CyI_90_ results presented in Figure 8, we reported the *p*-value of the one-way ANOVA (drug effect) for each cell lines. Idarubicin stood out as the anticancer-drug with the most significant different CyI_90_ in comparison with the other cytotoxic agents over Caco-2 (*p* < 0.001), HT29 (*p* < 0.001), SW480 (*p* = 0.020), and SW 620 (*p* < 0.001) (Figure 8a–d). For HCT 116, there was a trend for idarubicin to be the best candidate (*p* = 0.140) (Figure 8e). Gemcitabine had the highest CyI_90_ value for SW48, but without a significant difference (*p* = 0.490) (Figure 8f). The significant *p*-values of the post-hoc tests (LSD Fisher) were reported directly in Figure 8.

## 3. Discussion

Chemotherapeutic agents for IA treatment of CRLM are very heterogeneous. The rationale for drug choice has never been based on any cytotoxicity study. In the present study, we tested 12 anticancer drugs after short exposure time, in keeping with pharmacokinetics (PK) data observed during IA therapies [26], as suggested in other studies [27]. The main results of our study are that idarubicin emerged as the most cytotoxic agent to most of the studied CRC cell lines (Caco-2, HT29, HCT116, SW48, and SW480). Gemcitabine was the most efficient for SW48 (although statistically non-significant). Interestingly, the most commonly used cytotoxic agents in the systemic and intra-arterial treatment of CRLM (oxaliplatin, 5-FU, irinotecan) showed very limited cytotoxicity to all the tested cell lines.

The biphasic curves for anthracyclines and the cell growth obtained with low dose of paclitaxel may need an additional study on the mechanisms of response. Nevertheless, according to our results, paclitaxel did not seem to be significant over the CRC cell lines.

Boulin et al., who used a similar methodology (cytotoxicity evaluation after a 30-min exposure time) on 3 HCC cell lines, also found idarubicin to be the best cytotoxic agent for TACE in HCC treatment [27]. Idarubicin is an anthracycline interacting with topoisomerase II and inhibiting the synthesis of nucleic acids. Modification on Position 4 on anthracycline hub gives a strong lipophilicity to idarubicin, which leads to strong intracellular penetration, better than that of other anthracyclines. If we focus on TACE, idarubicin pharmacokinetics with lipiodol were evaluated in-vitro and in-vivo [32]. The hepatic extraction ratio of idarubicin has not been reported in the literature so far. However, a 40% biodisponibility of idarubicin was published after intra-arterial administration of idarubicin-TACE, thereby suggesting a favorable extraction ratio [32]. Idarubicin has been widely explored for IA treatment, either combined with lipiodol [33] or with drug-eluting beads [29,31]. In addition to these chemotherapies, we also tested lipiodol, one of the available vectors for intra-arterial therapy. No significant effect of lipiodol was reported on CRC cell line viability (Supplementary data), which showed that this drug could definitely be regarded as a vector without any efficacy by itself. Phase I studies with IA idarubicin exhibited high toxicity profile in human patients with HCC. Therefore, in terms of feasibility, the use of idarubicin-based regimen for IA treatment should be even better tolerated in CRLM patients, who are most likely less fragile than cirrhotic patients.

Response to standard oncologic treatment is limited in CRC and there is a great potential to improve treatment efficacy by the molecularly-guided repurposing of targeted drugs. De facto, molecular classification of CRC has evolved in recent years, resulting in the four biologically distinct consensus molecular subtypes (CMS): CMS1 MSI (microsatellite instability)-immune, CMS2 epithelial and canonical, CMS3 epithelial and metabolic, and CMS4 mesenchymal [34]. The CMS classification has prognostic value independent of cancer stage, with dismal survival outcomes for the CMS4 population [35]. In the metastatic setting, patients with MSI tumors have a poor prognosis, but respond well to immune checkpoint inhibition [36]. A potential predictive value of the CMS groups was also suggested from retrospective analyses of clinical trials, including lack of benefit from oxaliplatin and anti-EGFR treatment [35,37] in tumors with a mesenchymal-like phenotype, the latter being independent of RAS mutation status. However, increased understanding of the unique drug sensitivities of the individual CMS groups has great potential to advance precision medicine in colorectal cancer.

Caco-2, HCT 116, SW480, and SW620 belong to CMS 4 (i.e., the mesenchymal group), associated with the most aggressive disease and the worst survival. For all these CRC cell lines, idarubicin is the best candidate in our study. HT29 belongs to CMS 3, i.e., the metabolic group. Idarubicin is also the best candidate with a short exposure time. As for SW48 which is classified into CMS 1, i.e., an MSI-immune subtype, gemcitabine appears as the most interesting drug compared with other drugs. Our results are thus in line with those of Sveen et al. [38] who reported that the most efficient drugs for CMS1 and CMS4 cell lines were the inhibitors of topoisomerase II and of antimetabolite, especially idarubicin and gemcitabine [38]. In this substantial work, they also reported that idarubicin and gemcitabine had an effect over MSI+ cell lines. Again, our results were aligned with this study, despite the very short exposure time.

A comparison of the present results with the literature was carried out based on the few available studies. Three studies evaluated whether oxalipatin, irinotecan, and gemcitabine could have IC_50_ compatible with CIAH [39,40,41]. Only Hofmann et al. evaluated irinotecan efficacy on CRC cell lines with the same exposure time (30 min) [41]. Cytotoxicity evaluation used the HTCA (Human Tumor colony-forming assay). IC_50_ on HT29 was 200 µg/mL, which was similar to that obtained in the present study (202 ± 57 µg/mL). Kornmann et al. studied in-vitro effect of oxaliplatin and gemcitabine with a 2-h, 4-h, and 24-h exposure time on CRC cell lines (HT29, NMG64/84 colon and COLO 357) and on fresh liver metastases [39]. The respective IC_50_ values for oxaliplatin and gemcitabine were <10 µg/mL and 100 µg/mL after a 2-h exposure on HT29. In the present study, the IC_50_ values on HT29 after a 30-min exposure were 40.9 ± 41 µg/mL for oxaliplatin and 793 µg/mL for gemcitabine, which clearly highlighted the time-dependent effect of these two drugs. A concentration dependence is also known for gemcitabine [42]. No study has evaluated the IC_90_ of cytotoxic agents over CRC cell lines. Yet, the stronger the cytotoxicity index, the higher the chance to find an effective agent for locoregional therapies. In addition, colorectal adenocarcinomas are supposed to be sensitive to anthracyclines and other topoisomerase II inhibitors. The Caco-2 resistance was well studied and related to two mechanisms: MDR (Multi Drug Resistance) system and a confluence-dependent resistance [43]. Our results showed a significant efficacy of idarubicin over Caco-2, which is possibly correlated with the known characteristics of idarubicin to overcome glycoprotein (PgP)-related MDR [44].

Some limitations to our study must be acknowledged. First, the cell viability analysis methodology was based on WST-1, which is a metabolic marker less sensitive than crystal violet (cell marker) used by Boulin et al. [27]. However, WST-1 was chosen because it could provide a fast and sensitive evaluation of cell viability and proliferation for the 9504 culture wells analyzed. Moreover, to ensure robust results, four replicates per experiment were relied upon with three independent experiments for each condition. Second, we did not test any CMS 2 cell line. Nevertheless Sveen et al. found poor sensitivity to chemotherapy and, on the contrary, extreme sensitivity to anti EGFR and HERB2 [38]. Interestingly, we tested both wild-type and mutated RAS cell lines and the efficacy of idarubicin appeared to be independent of RAS status. Third, in this drug screening study of cell lines, Sveen et al. identified CMS1 and CMS4 as potential predictive biomarkers for response to HSP90 inhibition. In vivo, this targeted treatment may alleviate chemoresistance in CMS4. Another point is related to the characteristics of the studied cell lines with respect to Duke’s staging system. Only one tested cell line (HCT116) came from a Duke’s stage D adenocarcinoma. Nevertheless, to our knowledge, there are only three cell lines coming from a Duke’s stage D, i.e., with the ability to colonize the liver in clinical conditions. All these three cell lines are RAS-mutated, which explains why we only chose one.

Another perspective could be to test “combined chemotherapies”.

According to our results, we plan to propose a phase II trial with idarubicin in MSI-mCRC after failure of two systemic treatment lines.

## 4. Materials and Methods

### 4.1. Cell Lines

CRC is a heterogeneous disease mainly related to the heterogeneity of genomic instability. We chose different cell lines to represent the different molecular characteristics of CRC (BRAF and/or KRAS mutations, as well as BRAF/KRAS wt; Chromosomal Instability (CIN) or MicroSatellite Instability (MSI)) coming from primary or metastatic site and located at different places in the colon.

Six human CRC cell lines (Caco-2, HCT 116, HT 29, SW 48, SW 480, SW 620) were selected from the American Type Culture Collection (ATCC). Their characteristics and their respective CMS groups [34] are summarized in Table 4. Caco-2 cell lines were cultured without specific conditions, to keep the adenocarcinoma profile and not an intestinal epithelial barrier. Caco-2 has the advantage to be KRAS-wild type, to have a MDR system, and to come from the right colon. All the cell culture products were provided by Sigma-Aldrich (St-Quentin Fallavier, France), except for fetal bovine serum (FBS), which was purchased from Eurobio (Courtaboeuf, France). The cell lines were cultured at 37 °C in a humidified atmosphere in the presence of 5% CO_2_ in RPMI (Roswell Park Memorial Institute), 1640 were supplemented with 10% decomplemented FBS (20% FBS for Caco-2 cells), 1% L-glutamine and 1% penicilline-streptomicine. All cell lines were tested to be mycoplasma-free.

### 4.2. Chemotherapeutic Drugs

A total of 13 molecules were tested, currently used in CRC or not. Twelve cytotoxic agents, all of which are routinely used in colorectal cancer for systemic treatment (oxaliplatin, 5-FU, irinotecan, but also raltitrexed and paclitaxel used in case of contra-indications to fluoropyrimidine). We included chemotherapeutic agents used for intra-arterial treatments and idarubicin, the best candidate after in-vitro screening on HCC cell lines [27]. We selected (a) three anthracyclines: doxorubicin (200 mg/100 mL; Teva Sante, Courbevoie, France); idarubicin (10 mg/10 mL; Zavedos, Pfizer, Paris, France) ; epirubicin 50 mg/25 mL; Mylan, Paris, France), (b) one alkylating antibiotic (mitomycin C, 10 mg; Kyowa Kirin Pharma, Neuilly-Sur-Seine, France), (c) three anti-metabolites: 5-FU (5 g/100 mL; Accord Healthcare, Lille, France); gemcitabine (38 mg/mL; Hospira, Meudon, France); raltitrexed (2 mg; Sigma-Aldrich, Saint-Louis, MO, USA), (d) one DNA topoisomerase inhibitor: irinotecan (500 mg/25 mL; Hospira, Meudon, France), (e) two platinum derivatives: oxaliplatin (200 mg/40 mL; Accord Healthcare, Lille, France); cisplatin (100 mg/100 mL; Accord Healthcare, Lille, France), (f) one antitumoral antibiotic: streptozotocin (1 g; Zanozar, Keocyt, Montrouge, France) and (g) one taxane: paclitaxel (300 mg/50 mL; Hospira, Meudon, France). We also included the vector used for drug mixture in c-TACE: lipiodol (480 mg Iode/mL; Guerbet, Villepinte, France). Because raltitrexed was not available in the cytotoxic lab of the university hospital, this molecule was used from a pure chemical powder form, reconstituted in the research lab according to the manufacturer’s recommendations. The 12 other molecules we used, which were in their marketed pharmaceutical form, came from the cytotoxic lab of the university hosital in solution form (in sterile 10 mL syringes). Only two of them (mitomycin C and streptozocin) had beforehand been reconstituted from powder form in RPMI. All the solutions were used after warming at 37 °C. The maximal concentrations used (C Max) for the present study were: doxorubicin 2 mg/mL; idarubicin 1 mg/mL; epirubicin 2 mg/mL; mitomycin C 1 mg/mL; 5-FU 50 mg/mL; gemcitabine 38 mg/mL; raltitrexed 0.5 mg/mL; irinotecan 20 mg/mL; oxaliplatin 5 mg/mL; cisplatin 1 mg/mL; streptozotocin 200 mg/mL; paclitaxel 6 mg/mL; bevacizumab 25 mg/mL, and lipiodol 480 mg Iodine/mL.

### 4.3. Screening Protocol

All the manipulations were carried out by two independent operators with, respectively, 4 and 27 years of experience in cell cultures and experimental studies. Cell lines were detached (Day 0) with a mixture of 0.05% trypsine/0.5 mM EDTA (Sigma-Aldrich, Saint-Louis, MO, USA) after rinsing with Dulbecco’s Phosphate Buffered Saline (PBS). Cells were seeded onto a 96-well tissue culture-plate, 10,000 cells per well, and cultured for 24 h. At Day 1, the culture medium was changed to lower the FBS level to 0.5%. Drugs with the maximal concentration were deposited in the first column of the plate and we attended to successive three-fold dilutions with fresh RPMI containing 0.5% FBS. The last column (Column 12) of the plate contained drug-free medium. The cells were exposed to drugs for 30 min at 37 °C before being washed twice or thrice if the drugs were viscous or stained with RPMI (0.5% FBS). The cells were then put back in the incubator for 72 h. For each condition (type of molecule, concentration of molecule and cell line type), replicates (*n* = 4) were tested during the same experience. Each experience was repeated three times (*N* = 3), as previously described for a total period of four months.

### 4.4. Cytotoxicity Assay

At Day 4, to evaluate the effect of drugs on cell viability, we performed spectrophotometric quantification by using the WST-1 Cell Viability Assay (WST-1, Sigma-Aldrich, Saint-Louis, MO, USA). Ten µL WST-1 per well were added to 100 µL of medium. After 2 h of incubation at 37 °C, the plate was shacked and absorbance was read on a microplate reader (Asys Hitech, Eugendorf, Austria) at 440 nm incubation in a humidified atmosphere (37 °C, 5% CO_2_), as recommended by the WST-1manufacturer.

### 4.5. Statistical and Data Analysis

The mean ± standard deviation of the repetition (*N* = 3) obtained from the mean of each quadruplicates was calculated. After normalization with the control (100% of viability), the viability curves were generated for the six cell lines treated by each of the 12 drugs, the lipiodol and the bevacizumab (Figure 1, Figure 2, Figure 3, Figure 4, Figure 5, Figure 6, Figure 7 and Figure 8). On each curve, a horizontal reference line corresponding to 10% of cell viability was represented.

The dose-response data were fitted by a four-parameter logistic nonlinear regression model (Equation (1)), modified from the original Hill equation [46]

(1) $$\frac{OD\left(c\right)-OD_{baseline}\left(c\right)}{OD\left(0\right)}=\alpha +\frac{1-\beta }{1+{\frac{c}{\gamma }}^{\delta }},$$

where c is the drug concentration, OD(c) is the optic density (OD) at the concentration of drug c, $OD_{baseline}\left(c\right)$ is the optic density of the rpmi and drug only and (α, β, γ, δ) are the four estimated parameters of the dose-response model. The Raltitrexed drug showed a different dose response pattern and was fitted using a biphasic dose response curve corresponding to a balanced sum of two Hill functions. The Matlab (MathWorks^®^; R2018b; Natick, MA, USA) non-linear solver (Curve Fitting Toolbox) estimated the parameters of the dose response function for each of the cell lines and drug conditions. Based on the fitted curve, we extracted the IC_50_ and IC_90_, which correspond to the drug concentration (mg/mL) required to obtain 50% and 90% of cell death, respectively. Finally, we calculated the CyI_90_ cytotoxicity index_,_ corresponding to the ratio of the maximal drug concentration to IC_90_ (CyI_90_ = C Max/IC_90_), according to Boulin et al. [27]. For example, a CyI_90_ of 1000 means that the drug kills 90% of the cells even when diluted (1:1000). The IC_50_, IC_90,_ and CyI_90_ values are represented in Table 1, Table 2 and Table 3. To compare each molecule effect on each cell line, the mean of CyI_90_ was tested with a one-way ANOVA. Whenever there was a significant mean difference between the drugs, an additional post-hoc test (Fisher’s LSD) was conducted. A *p* value < 0.05 was considered statistically significant. Results were reported as mean ± standard deviation for each cell lines.

All the statistical analyses were performed with Statistica (TIBCO^®^ Software; 13.4.0.14; Palo Alto, CA, USA).

## 5. Conclusions

The most commonly used anticancer drugs (5-FU, oxaliplatin, irinotecan, raltitrexed) in CRC have a very limited cytotoxicity effect on CRC cell lines after short exposure time (30 min). On the contrary, idarubicin (and partially gemcitabine) exhibits a strong cytotoxicity profile (over CMS1, 3, 4 cell lines, independently of RAS status). These results argue for further trials of idarubicin (optionally combined with gemcitabine) for the intra-arterial treatment of CRC liver metastases in patients with unresectable CRLM. In the same way, the poor patient prognosis associated with CMS4 warrants additional studies to pursue the potential for clinical testing of HSP90 inhibitor repositioning and combination therapy with idarubicin in colorectal cancer.

## Figures and Tables

**Table 1:** IC_50_ values (mean ± standard deviation) obtained for 6 colorectal cancer cell lines and 12 antitumoral agents. The bold is to underline the name of the drug, distinguish from the family of the drug.

	Cell Line	Caco-2	HCT116	HT29	SW48	SW480	SW620
Drug							
Topoisomerase II inhibitor **doxorubicin**		0.22 ± 0.28	0.01 ± 0.01	0.001 ± 0.001	0.0007 ± 0.0006	0.0006 ± 0.0003	0.0008 ± 0.0007
Topoisomerase II inhibitor **epirubicin**		0.03 ± 0.02	0.01 ± 0.01	0.001 ± 0.001	0.0007 ± 0.0001	0.0003 ± 0.0002	0.0005 ± 0.0002
Topoisomerase II inhibitor **idarubicin**		0.0004 ± 0.0001	0.00005 ± 0.00006	0.00003 ± 0.00002	0.00002 ± 0.00001	0.00007 ± 0.00009	0.00003 ± 0.00002
Anti-metabolites **5-FU**		0.47 ± 0.08	213.75 ± 345.11	0.007 ± 0.001	0.01 ± 0.005	0.02 ± 0.01	0.01 ± 0.009
Anti-metabolites **raltitrexed**		0.25 ± 0.07	0.08 ± 0.08	0.12 ± 0.22	0.10 ± 0.17	0.05 ± 0.08	0.0002 ± 0.0002
Anti-metabolites **gemcitabin**		3.03 ± 2.21	0.09 ± 0.15	0.79 ± 1.36	0.0004 ± 0.0006	0.05 ± 0.09	0.0001 ± 0.00005
Platinum derivatives **cisplatin**		0.09 ± 0.10	0.14 ± 0.03	0.01 ± 0.005	0.01 ± 0.01	0.01 ± 0.003	0.01 ± 0.007
Platinum derivatives **oxaliplatin**		0.02 ± 0.01	0.61 ± 00.39	0.04 ± 0.04	0.04 ± 0.06	0.07 ± 0.06	0.008 ± 0.005
Alkylating antibiotic **mitomycin C**		0.08 ± 0.08	0.02 ± 0.01	0.005 ± 0.007	0.001 ± 0.0008	0.004 ± 0.005	0.002 ± 0.0007
Topoisomerase I inhibitor **irinotecan**		0.95 ± 0.83	0.20 ± 0.13	0.20 ± 0.05	0.07 ± 0.09	0.12 ± 0.10	0.18 ± 0.10
Antitumoral antibiotic **streptozocin**		22.93 ± 10.86	24.58 ± 1.33	19.31 ± 10.15	13.88 ± 3.21	18.09 ± 4.77	2.65 ± 1.12
Taxane **paclitaxel**		0.10 ± 0.03	0.05 ± 0.09	0.25 ± 0.19	0.0006 ± 0.0009	0.16 ± 0.22	0.05 ± 0.05

**Table 2:** IC_90_ values (mean ± standard deviation) obtained for the 6 colorectal cancer cell lines and the 12 antitumoral agents. The bold is to underline the name of the drug, distinguish from the family of the drug.

	Cell Line	Caco-2	HCT116	HT29	SW48	SW480	SW620
Drug							
Topoisomerase II inhibitor **doxorubicin**		2.68 ± 2.02	5.40 ± 4.77	1.47 ± 1.47	0.07 ± 0.10	0.13 ± 0.08	0.02 ± 0.009
Topoisomerase II inhibitor **epirubicin**		1.55 ± 0.93	4.16 ± 4.07	na	0.10 ± 0.02	0.43 ± 0.36	0.04 ± 0.03
Topoisomerase II inhibitor **idarubicin**		0.009 ± 0.007	0.04 ± 0.04	0.0005 ± 0.0003	0.002 ± 0.001	0.002 ± 0.001	0.0003 ± 0.0001
Anti-metabolites **5-FU**		138.28 ± 193.78	82,843.1 ± 103,130.2	1.31 ± na	2.97 ± 4.74	1.92 ± 2.75	1.77 ± 0.27
Anti-metabolites **raltitrexed**		260.88 ± 36.32	221.28 ± 12.01	173.89 ± 91.41	181.32 ± 20.44	202.82 ± 8.82	538.65 ± 437.50
Anti-metabolites **gemcitabin**		202.35 ± 284.002	55.87 ± 37.11	9832.8 ± 13,788.9	3.87 ± 4.36	77.70 ± 110.10	0.74 ± 0.40
Platinum derivatives **cisplatin**		2.81 ± 2.47	2.53 ± 2.03	3.34 ± 4.26	00.50 ± 0.79	0.87 ± 0.68	0.20 ± 0.11
Platinum derivatives **oxaliplatin**		0.40 ± 0.23	5.57 ± 3.59	2.24 ± 1.64	0.83 ± 1.20	1.34 ± 0.79	0.26 ± 0.26
Alkylating antibiotic **mitomycin C**		0.80 ± 0.76	0.45 ± Na	0.08 ± 0.08	0.04 ± 0.05	0.13 ± 0.14	0.04 ± 0.03
Topoisomerase I inhibitor **irinotecan**		8.91 ± 7.10	2.39 ± 0.86	13.64 ± 10.02	15.34 ± 11.25	11.28 ± 5.57	19.77 ± 27.26
Antitumoral antibiotic **streptozocin**		20.70 ± 0.54	0.88 ± 0.57	20.03 ± 10.35	10.31 ± 10.33	0.66 ± 0.64	0.20 ± 0.15
Taxane **paclitaxel**		1.99 ± 1.72	3.02 ± 2.60	1.73 ± 1.79	585.43 ± 1011.63	1.56 ± 1.98	41.65 ± 50.47

**Table 3:** Cytotoxic Index (CyI_90_) values (mean ± standard deviation) obtained for the 6 colorectal cancer cell lines and the 12 antitumoral agents. The bold is to underline the name of the drug, distinguish from the family of the drug. Numbers in bold are the highest values for each column (cell line type).

	Cell Line	CaCo2	HCT116	HT29	SW48	SW480	SW620
Drug							
Topoisomerase II inhibitor **doxorubicin**		1.13 ± 0.84	0.76 ± 0.79	2.72 ± 2.72	359.57 ± 556.29	25.30 ± 25.53	96.25 ± 31.85
Topoisomerase II inhibitor **epirubicin**		1.58 ± 0.74	0.92 ± 0.90	na ± na	19.90 ± 4.08	7.25 ± 6.19	61.78 ± 35.10
Topoisomerase II inhibitor **idarubicin**		**156.97 ± 114.03**	**83.32 ± 99.27**	**2170.84 ± 993.71**	468.19 ± 223.29	**479.79 ± 235.56**	**3168.35 ± 1567.32**
Anti-metabolites **5-FU**		1.37 ± 1.14	0.94 ± 1.26	37.92 ± na	276.55 ± 368.76	191.74 ± 250.78	28.53 ± 4.47
Anti-metabolites **raltitrexed**		0.19 ± 0.04	0.72 ± 0.37	0.36 ± 0.27	1.01 ± 1.14	1.31 ± 0.93	4.45 ± 4.48
Anti-metabolites **gemcitabin**		0.70 ± 0.56	0.86 ± 0.42	0.23 ± 0.32	**18,126.37 ± 31,381.13**	252.01 ± 435.20	71.45 ± 56.09
Platinum derivatives **cisplatin**		0.62 ± 0.50	0.56 ± 0.31	1.59 ± 2.02	15.46 ± 14.33	1.59 ± 0.86	6.45 ± 4.15
Platinum derivatives **oxaliplatin**		14.78 ± 6.91	1.30 ± 1.00	4.36 ± 4.62	658.91 ± 1124.13	5.67 ± 5.01	45.61 ± 47.78
Alkylating antibiotic **mitomycin C**		2.00 ± 1.24	2.18 ± na	19.86 ± 12.45	125.52 ± 203.90	13.60 ± 9.02	34.54 ± 20.12
Topoisomerase I inhibitor **irinotecan**		3.60 ± 2.84	9.12 ± 3.31	2.83 ± 2.98	1.78 ± 1.31	2.15 ± 1.20	3.68 ± 3.18
Antitumoral antibiotic **streptozocin**		0.77 ± 0.11	0.91 ± 0.05	1.49 ± 1.00	1.11 ± 0.13	0.99 ± 0.04	0.55 ± 0.45
Taxane **paclitaxel**		5.12 ± 3.54	5.77 ± 7.37	6.76 ± 5.18	7.68 ± 11.24	30.66 ± 48.10	3.84 ± 4.66

**Table 4:** Characteristics of the six colorectal cancer (CRC) cell lines used in the study, including molecular characteristics (mutations are annotated at the protein level, as described by den Dunnen et al. [45]; wt: wild type) and the relevant consensus molecular subtypes (CMS) groups according to Sveen et al. [38].

CRC Cell Line Designation	Caco-2	HCT116	HT-29	SW48	SW480	SW620
(ATCC Number)	(HTB-37)	(CCL-247)	(HTB-38)	(CCL-231)	(CCL-228)	(CCL-227)
**Image (Day 1; X20)**	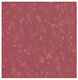	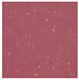	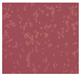	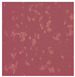	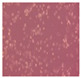	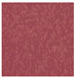
**Disease**	colorectal carcinoma	colorectal carcinoma	colorectal adenocarcinoma	colorectal adenocarcinoma	colorectal adenocarcinoma	colorectal adenocarcinoma
**Primary tissue**	colon	colon	colon	colon	colon	colon
**Tumor localization**	colon	ascending colon	colon	transverse colon	descending colon	descending colon
**Duke’stype**	n/a	D	C	C	B	C
**Grade**	n/a	n/a	n/a	IV	n/a	n/a
**Metastatic site**						lymph node
**Patient age (yo)**	72	48	44	83	50	51
**Patient gender**	male	male	female	female	male	male
**Ethnicity**	caucasian	na	caucasian	caucasian	caucasian	caucasian
**Genes expressed**	EGF	CEA	CEA	CEA	EGF	CEA
**TP53**	E204X	wt	R273H	wt	R273H;P309S	R273H;P309S
**K-ras**	wt	G13D	wt	wt	G12V	G12V
**B-raf**	wt	wt	V600E	wt	wt	wt
**PTEN**	wt	wt	Wt	wt	wt	wt
**PIK3CA**	wt	H1047R	P449T	wt	wt	wt
**CIN**	x		X		x	X
**MSS/MSI**	MSS	MSI	MSS	MSI	MSS	MSS
**CMS**	4	4	3	1	4	4

## Data Availability

The data presented in this study are available in the main text and supplementary material.

## Supplementary Materials

The following are available online at https://www.mdpi.com/article/10.3390/ph14070639/s1, Figure S1: Colorectal cancer cell line viability curves after a 30-min time-contact with lipiodol. The horizontal red line represents 10% of cell viability. Table S1: Statistical analysis (LSD-Fisher) for cell viability curves. Values represents the LSD-Fisher p-value for any concentration versus control, for each drug and each cell line.
