# Supplementary material for: Anticancer Drugs for Intra-Arterial Treatment of Colorectal Cancer Liver Metastases: In-Vitro Screening after Short Exposure Time
Source: Pharmaceuticals (Basel). 2021 Jul 1;14(7):639. doi: 10.3390/ph14070639 (PMC8308869; doi:10.3390/ph14070639)
Supplement: Supplementary file 1 [file pharmaceuticals-14-00639-s001.zip › pharmaceuticals-1285237-supplementary.pdf]

Supplementary Material:

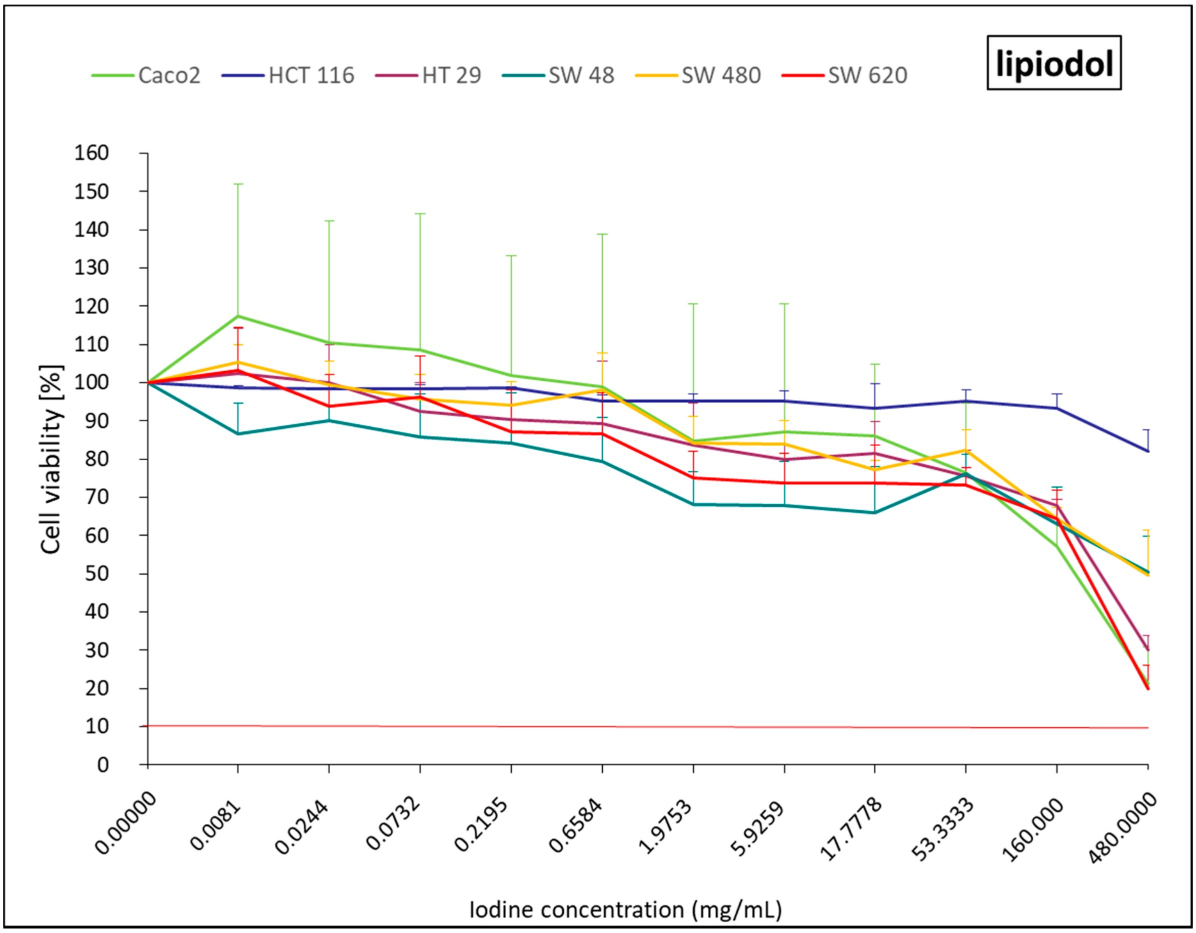

Figure S1. Colorectal cancer cell line viability curves after a 30-min time-contact with lipiodol. The horizontal red line represents 10% of cell viability. The results are presented after normalization with the control cells (untreated condition).

Table S1. Statistical analysis (LSD-Fisher) for cell viability curves. Values represents the LSD-Fisher p-value for any concentration versus control, for each drug and each cell line. P-value <0.05 are in red.

	doxorubicin					
	CaCO2	HCT 116	HT29	SW48	SW480	SW620
C Max / 59 049	0.783576	0.180173	0.276931	0.096282	0.000682	0.033041
C Max / 19 683	0.562118	0.044211	0.009418	0.008290	0.000001	0.000083
C Max / 6561	0.923713	0.165543	0.000331	0.000473	0.000000	0.000005
C Max / 2187	0.899942	0.000419	0.000002	0.000063	0.000000	0.000000
C Max / 729	0.895942	0.000000	0.000000	0.000023	0.000000	0.000000
C Max / 243	0.676869	0.000000	0.000000	0.000000	0.000000	0.000000
C Max / 81	0.121786	0.000000	0.000000	0.000000	0.000000	0.000000
C Max / 27	0.019697	0.000000	0.000000	0.000001	0.000000	0.000000
C Max / 9	0.001408	0.000000	0.000000	0.000000	0.000000	0.000000
C Max / 3	0.000279	0.000000	0.000000	0.000000	0.000000	0.000000
C Max	0.000001	0.000000	0.000000	0.000000	0.000000	0.000000
	epirubicin					
	CaCO2	HCT 116	HT29	SW48	SW480	SW620
C Max / 59 049	0.878195	0.214789	0.607786	0.072010	0.006276	0.000004
C Max / 19 683	0.946534	0.242493	0.061231	0.005525	0.000007	0.000000
C Max / 6561	0.379920	0.266857	0.003066	0.000000	0.000000	0.000000
C Max / 2187	0.171299	0.000166	0.000032	0.000000	0.000000	0.000000
C Max / 729	0.013034	0.000000	0.000000	0.000000	0.000000	0.000000
C Max / 243	0.000259	0.000000	0.000000	0.000000	0.000000	0.000000
C Max / 81	0.000010	0.000000	0.000000	0.000000	0.000000	0.000000
C Max / 27	0.000000	0.000000	0.000000	0.000000	0.000000	0.000000
C Max / 9	0.000000	0.000000	0.000000	0.000000	0.000000	0.000000
C Max / 3	0.000000	0.000000	0.000000	0.000000	0.000000	0.000000
C Max	0.000000	0.000000	0.000000	0.000000	0.000000	0.000000
	idarubicin					
	CaCO2	HCT 116	HT29	SW48	SW480	SW620
C Max / 59 049	0.720627	0.000000	0.529328	0.002716	0.732430	0.047629
C Max / 19 683	0.285238	0.000000	0.056943	0.001411	0.984214	0.000000
C Max / 6561	0.002090	0.000000	0.000734	0.000502	0.680906	0.000000
C Max / 2187	0.000000	0.000000	0.000083	0.000012	0.034879	0.000000
C Max / 729	0.000000	0.000000	0.000073	0.000020	0.012891	0.000000
C Max / 243	0.000000	0.000000	0.007693	0.000042	0.189255	0.000000
C Max / 81	0.000000	0.000000	0.001307	0.000212	0.080015	0.000000
C Max / 27	0.000000	0.000000	0.000017	0.000008	0.008365	0.000000
C Max / 9	0.000000	0.000000	0.000012	0.000006	0.005906	0.000000
C Max / 3	0.000000	0.000000	0.000018	0.000005	0.007215	0.000000
C Max	0.000000	0.000000	0.000015	0.000113	0.010214	0.000000
	5-FU					
	CaCO2	HCT 116	HT29	SW48	SW480	SW620
C Max / 59 049	0.588898	0.955069	0.033774	0.206785	0.542621	0.959883
C Max / 19 683	0.674734	0.993181	0.000009	0.066833	0.110778	0.000021
C Max / 6561	0.143048	0.970795	0.000000	0.000010	0.000056	0.000012
C Max / 2187	0.007930	0.530379	0.000000	0.000000	0.000000	0.000001

C Max / 729	0.000724	0.188069	0.000000	0.000000	0.000000	0.000000
C Max / 243	0.000027	0.454271	0.000000	0.000000	0.000000	0.000000
C Max / 81	0.000001	0.165215	0.000000	0.000000	0.000000	0.000000
C Max / 27	0.000000	0.014706	0.000000	0.000000	0.000000	0.000000
C Max / 9	0.000000	0.000764	0.000000	0.000000	0.000000	0.000000
C Max / 3	0.000000	0.000064	0.000000	0.000000	0.000000	0.000000
C Max	0.000000	0.000000	0.000000	0.000000	0.000000	0.000000
	**raltitrexed**					
	**CaCO2**	**HCT 116**	**HT29**	**SW48**	**SW480**	**SW620**
C Max / 59 049	0.104017	0.999016	0.829700	0.911253	0.856710	0.361116
C Max / 19 683	0.107258	0.816367	0.878751	0.985889	0.893396	0.075492
C Max / 6561	0.541102	0.888802	0.800379	0.110970	0.702164	0.002447
C Max / 2187	0.369618	0.820971	0.346700	0.000476	0.184678	0.000165
C Max / 729	0.972874	0.402383	0.205667	0.000130	0.080757	0.000055
C Max / 243	0.250864	0.392428	0.161153	0.002552	0.153314	0.000001
C Max / 81	0.855081	0.177644	0.165215	0.000342	0.066727	0.000001
C Max / 27	0.701725	0.009413	0.092201	0.000141	0.040921	0.000000
C Max / 9	0.318693	0.004038	0.220232	0.000135	0.030790	0.000000
C Max / 3	0.005422	0.000626	0.236073	0.000083	0.012613	0.000000
C Max	0.000000	0.000000	0.000698	0.000005	0.000762	0.000000
	**gemcitabine**					
	**CaCO2**	**HCT 116**	**HT29**	**SW48**	**SW480**	**SW620**
C Max / 59 049	0.065324	0.012074	0.052981	0.000022	0.010950	0.00
C Max / 19 683	0.312947	0.005948	0.068738	0.000010	0.043549	0.00
C Max / 6561	0.511429	0.006540	0.049740	0.000005	0.015645	0.00
C Max / 2187	0.333330	0.000842	0.046894	0.000001	0.001154	0.00
C Max / 729	0.209661	0.000066	0.024041	0.000001	0.000807	0.00
C Max / 243	0.086645	0.000037	0.020372	0.000000	0.000644	0.00
C Max / 81	0.070336	0.000010	0.020149	0.000000	0.000445	0.00
C Max / 27	0.042973	0.000006	0.021192	0.000000	0.000329	0.00
C Max / 9	0.007329	0.000002	0.013916	0.000000	0.000141	0.00
C Max / 3	0.000000	0.000000	0.000146	0.000000	0.000012	0.00
C Max	0.000000	0.000000	0.000054	0.000000	0.000013	0.00
	**cisplatin**					
	**CaCO2**	**HCT 116**	**HT29**	**SW48**	**SW480**	**SW620**
C Max / 59 049	0.909528	0.915765	0.979775	0.817637	0.150723	0.867400
C Max / 19 683	0.845843	0.823523	0.912750	0.419344	0.288074	0.808878
C Max / 6561	0.997337	0.981214	0.538824	0.294622	0.613341	0.139744
C Max / 2187	0.769288	0.753844	0.629160	0.112114	0.103623	0.163361
C Max / 729	0.772946	0.781347	0.023687	0.461584	0.000645	0.095342
C Max / 243	0.483939	0.482962	0.002191	0.004189	0.000012	0.000029
C Max / 81	0.339277	0.387137	0.000000	0.000191	0.000000	0.000000
C Max / 27	0.005131	0.000016	0.000000	0.000015	0.000000	0.000000
C Max / 9	0.000049	0.000000	0.000000	0.000002	0.000000	0.000000
C Max / 3	0.000003	0.000000	0.000000	0.000001	0.000000	0.000000
C Max	0.000000	0.000000	0.000000	0.000001	0.000000	0.000000
	**oxaliplatin**					
	**CaCO2**	**HCT 116**	**HT29**	**SW48**	**SW480**	**SW620**
C Max / 59 049	0.762307	0.840433	0.674313	0.628147	0.665114	0.938514
C Max / 19 683	0.814301	0.959414	0.603271	0.433616	0.996916	0.543320
C Max / 6561	0.572786	0.928276	0.905187	0.315179	0.712286	0.267300
C Max / 2187	0.255470	0.650532	0.013333	0.100569	0.558075	0.000033

C Max / 729	0.067708	0.848791	0.002818	0.022747	0.001084	0.000000
C Max / 243	0.001305	0.437154	0.000068	0.016278	0.004190	0.000000
C Max / 81	0.000106	0.421409	0.000016	0.008482	0.000120	0.000000
C Max / 27	0.000010	0.049870	0.000000	0.001596	0.000001	0.000000
C Max / 9	0.000012	0.000129	0.000000	0.000773	0.000000	0.000000
C Max / 3	0.000003	0.000000	0.000000	0.000598	0.000000	0.000000
C Max	0.000001	0.000000	0.000000	0.000293	0.000000	0.000000

	**mitomycin c**					
	**CaCO2**	**HCT 116**	**HT29**	**SW48**	**SW480**	**SW620**
C Max / 59 049	0.691972	0.914507	0.710627	0.892610	0.501034	0.271583
C Max / 19 683	0.940662	0.859913	0.715526	0.774722	0.307664	0.118708
C Max / 6561	0.807983	0.803137	0.845628	0.047691	0.192476	0.002914
C Max / 2187	0.999702	0.568554	0.421334	0.000894	0.063375	0.000203
C Max / 729	0.758469	0.131871	0.075168	0.000060	0.001519	0.000000
C Max / 243	0.700307	0.000487	0.001491	0.000001	0.000027	0.000000
C Max / 81	0.172426	0.000000	0.000080	0.000000	0.000001	0.000000
C Max / 27	0.000675	0.000000	0.000007	0.000000	0.000000	0.000000
C Max / 9	0.000001	0.000000	0.000003	0.000000	0.000000	0.000000
C Max / 3	0.000000	0.000000	0.000003	0.000000	0.000000	0.000000
C Max	0.000000	0.000000	0.000001	0.000000	0.000000	0.000000

	**irinotecan**					
	**CaCO2**	**HCT 116**	**HT29**	**SW48**	**SW480**	**SW620**
C Max / 59 049	0.531639	0.798530	0.389022	0.498134	0.853986	0.970194
C Max / 19 683	0.817954	0.733610	0.923159	0.452620	0.226172	0.072380
C Max / 6561	0.873064	0.947787	0.221012	0.072979	0.460960	0.637463
C Max / 2187	0.659807	0.549807	0.229030	0.004467	0.043006	0.383565
C Max / 729	0.882144	0.155519	0.023224	0.000683	0.006200	0.127432
C Max / 243	0.429677	0.001119	0.001581	0.000064	0.000167	0.003251
C Max / 81	0.138901	0.000000	0.000323	0.000022	0.000035	0.001487
C Max / 27	0.003098	0.000000	0.000000	0.000002	0.000004	0.000020
C Max / 9	0.000001	0.000000	0.000000	0.000001	0.000000	0.000001
C Max / 3	0.000001	0.000000	0.000000	0.000000	0.000000	0.000001
C Max	0.000001	0.000000	0.000000	0.000000	0.000000	0.000000

	**streptozocin**					
	**CaCO2**	**HCT 116**	**HT29**	**SW48**	**SW480**	**SW620**
C Max / 59 049	0.901238	0.971391	0.421162	0.884789	0.698013	0.843329
C Max / 19 683	0.965997	0.915907	0.629827	0.450871	0.840989	0.538511
C Max / 6561	0.365445	0.699865	0.923509	0.787214	0.945067	0.427658
C Max / 2187	0.819338	0.878870	0.656708	0.710166	0.684365	0.004309
C Max / 729	0.368347	0.797660	0.890968	0.350626	0.201647	0.000052
C Max / 243	0.274797	0.214547	0.255011	0.101211	0.318057	0.000000
C Max / 81	0.115040	0.054057	0.144423	0.001843	0.056790	0.000001
C Max / 27	0.046604	0.003801	0.000978	0.000290	0.008125	0.000000
C Max / 9	0.002518	0.000253	0.000034	0.000013	0.005607	0.000000
C Max / 3	0.000000	0.000000	0.000000	0.000000	0.000000	0.000000
C Max	0.000000	0.000000	0.000000	0.000000	0.000000	0.000000

	**paclitaxel**					
	**CaCO2**	**HCT 116**	**HT29**	**SW48**	**SW480**	**SW620**
C Max / 59 049	0.565191	0.009566	0.408540	0.859325	0.722051	0.534921
C Max / 19 683	0.364147	0.014782	0.907698	0.137379	0.928796	0.279793
C Max / 6561	0.083295	0.003726	0.911025	0.113074	0.915083	0.619407
C Max / 2187	0.020097	0.016540	0.251538	0.148185	0.844731	0.647216

C Max / 729	0.097149	0.042329	0.145584	0.939745	0.360457	0.942764
C Max / 243	0.034851	0.003338	0.357841	0.786418	0.019519	0.512017
C Max / 81	0.008287	0.000274	0.198257	0.411041	0.520269	0.097827
C Max / 27	0.000000	0.000001	0.036192	0.015631	0.294273	0.000823
C Max / 9	0.000000	0.000000	0.000486	0.012531	0.155020	0.001005
C Max / 3	0.000000	0.000000	0.000176	0.103943	0.130981	0.000127
C Max	0.000000	0.000000	0.000071	0.111858	0.082116	0.000067
